# Diagnosis of Appendicitis in Patients with Abnormal Position of the Appendix due to Mobile Caecum

**DOI:** 10.1155/2012/921382

**Published:** 2012-11-19

**Authors:** Huseyin Toprak, Mehmet Bilgin, Musa Atay, Ercan Kocakoc

**Affiliations:** Department of Radiology, Faculty of Medicine, Bezmialem Vakif University, Adnan Menderes Bulvari, Vatan Caddesi, Fatih, 34093 Istanbul, Turkey

## Abstract

Acute appendicitis is usually diagnosed on the basis of signs, symptoms, clinical history, physical examination, and results of laboratory tests.The position of the appendix can vary considerably, both in relation to the caecum and because of the inconsistent position of the caecum itself, and may cause variable clinical symptoms. We present the CT findings of surgically proven acute appendicitis associated with atypically located caecum in two patients.

## 1. Introduction

Acute appendicitis is usually diagnosed on the basis of signs, symptoms, clinical history, physical examination, and results of laboratory tests. However, in patients with atypical clinical features, due to abnormal position of the appendix, imaging studies play an important role in preoperative diagnosis and determination of appropriate treatment. The position of the appendix can vary considerably, both in relation to the caecum and because of the inconsistent position of the caecum itself. The mobile caecum and ascending colon are rare congenital anomalies. Embryologically, the caecum and ascending colon are usually covered retroperitoneally by the posterior peritoneum. If this normal process altered or stopped, the caecum and ascending colon may be suspended on a mesentery that allows the colon to move freely [1, 2]. Therefore, it is important to identify the caecum on CT in patients with suspicion of acute appendicitis so that an abnormal appendiceal location will be recognized. The aim of this paper is to present the CT findings of acute appendicitis associated with atypically located caecum in two patients.

## 2. Case Report

### 2.1. Case  1

**Figure 1:**
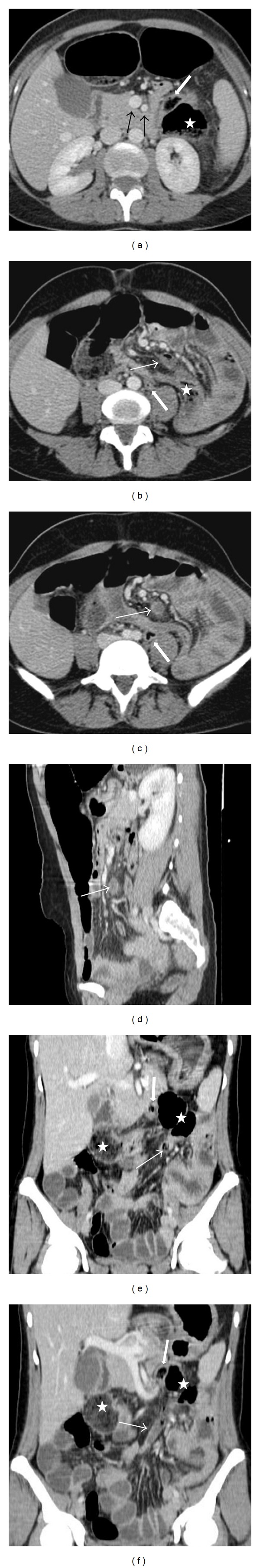
Axial (a–c), sagital (d) and coronal (e, f) CT images demonstrate absence of caecum and right hemicolon in the right side of abdomen. Normal position of superior mesenteric artery and vein (black arrows), so intestinal malrotation was excluded (a). Caecum and right hemicolon (star) located in the left upper quadrant adjacent to splenic flexure and descending colon (white closed arrows). CT images demonstrate 15 mm diameter tubular structure consistent with appendicitis located posteroinferior to the caecum, extending inferomedially to the umbilicus (white open arrows).

A 32-year-old woman was admitted to our hospital because of abdominal pain of one-day duration. The pain was localized in whole abdomen, constant and nonradiating. No other associated symptoms were noted. Physical examination revealed marked tenderness in the left upper and lower quadrant with moderate voluntary guarding. Her laboratory tests were normal except elevated white blood cell count of 15500/mL. Emergency US examination revealed normal uterus and bilateral ovaries, no signs of appendicitis in right lower quadrant, and colonic wall thickening. US revealed minimal free fluid in rectovesical pouch. US reported as normal by sonographer. Then, abdominopelvic CT examination with intravenous contrast material was performed (Asteion4-detector CT, Toshiba, Tokyo, Japan) to rule out other pathologies. Axial CT images demonstrate absence of caecum and right hemicolon in the right side of abdomen, normal position of superior mesenteric artery and vein (Figure 1(a)), so intestinal malrotation was excluded. Caecum and right hemicolon were located in the left upper quadrant adjacent to splenic flexure and descending colon (Figures 1(b) and 1(c)). Consecutive axial and coronal CT images demonstrate 15 mm diameter tubular structure consistent with appendicitis located posteroinferior to the caecum, extending inferomedially to the umbilicus (Figures 1(d), 1(e), and 1(f)). 

Surgery was performed under the general anesthesia with median incision extending from epigastrium to the suprapubic region. Operative findings included that right hemicolon was mobile till to the transverse colon and located in the left upper quadrant adjacent to splenic flexure and descending colon. The gangrenous appendix was found posteroinferior to the caecum, extending inferomedially to the umbilicus. Emergency appendectomy was performed, gangrenous appendix was proved pathologically, and she was discharged 3 days later after an uneventful recovery. 

### 2.2. Case  2

**Figure 2:**
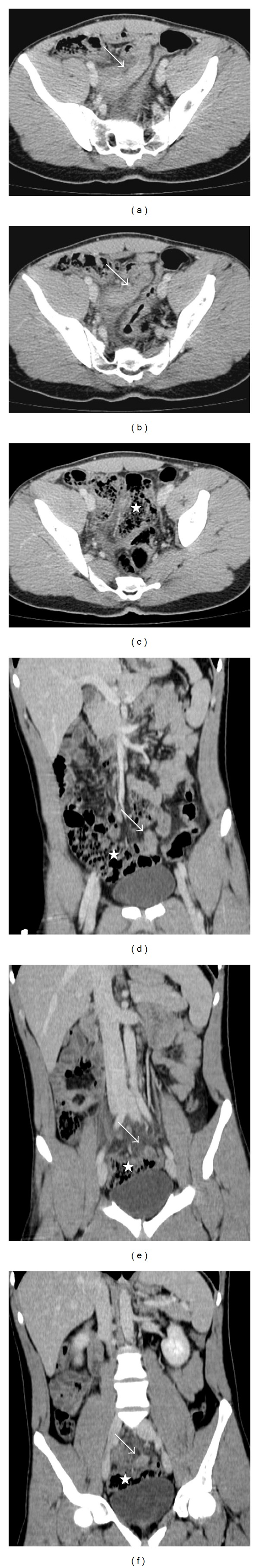
Axial (a–c) and coronal (d–f) CT images demonstrate pelvic location of the caecum (star). 3 consecutive axial and coronal CT images demonstrate 16 mm diameter tubular structure posterior to the caecum surrounded by inflamed mesenteric fat consistent with appendicitis (white arrows).

A 21-year-old man was admitted to our hospital because of abdominal pain of 12-hour duration. The pain was localized in the suprapubic region, persistent and noncolicky. No other associated symptoms were noted. Physical examination revealed mild tenderness in the right lower quadrant and suprapubic region with mild voluntary guarding. His laboratory tests were normal. Abdominopelvic CT examination with intravenous contrast material was performed (Asteion 4-detector CT, Toshiba, Tokyo, Japan) to rule out genitourinary and gastrointestinal pathologies. Axial and coronal CT images demonstrate pelvic location of the caecum and 16 mm diameter tubular structure posterior to the caecum surrounded by inflamed mesenteric fat consistent with appendicitis (Figures 2(a), 2(b), 2(c), 2(d), 2(e), and 2(f)). The base of the appendix is located in retrovesical pouch. The appendix extended laterally from retrovesical pouch to the right lower quadrant with the tip pointing towards the iliac vessels.

Surgery was performed under the general anesthesia with McBurney incision. Operative findings revealed that caecum was mobile and located in the pelvis. The base of the appendix located retrovesically. The appendix extended laterally from retrovesical pouch to the right lower quadrant with the tip pointing towards the iliac vessels. Emergency appendectomy was performed, phlegmonous appendix was proved pathologically, and he was discharged 2 days later after an uneventful recovery. 

## 3. Discussion

The incidence of mobile caecum and ascending colon has been estimated as 10–20% in literature [3, 4]. Despite this high incidence in the population, mobile caecum and ascending colon are very rare causes of acute abdomen [3, 4].

Embryologically, the cause of this abnormality is the result of the failure of fusion of right colonic mesentery with the lateral peritoneum. As a result, the caecum and ascending colon remain unattached to the posterior abdomen and are free to move and rotate. But, they usually reside in the right part of the abdomen [5].

Barium studies may be used to demonstrate abnormally located caecum and ascending colon. But, in patients with acute abdomen symptoms barium studies may delay the diagnosis and may lead to peritonitis in the case of perforation. US examination has a high sensitivity and specificity in the diagnosis of acute appendicitis. With US, radiologists can show inflamed appendix, abscess formation, free fluid and inflamed mesenteric fat. However, in the case of mobile caecum and ascending colon, US may lead to wrong diagnoses such as sigmoid diverticulitis, tuboovarian abscess, infected urachal, mesenteric or duplication cyst and Meckel diverticulitis [6]. In the case of normal appendix could not be demonstrated with US as in our first case, CT should be considered for diagnosis in spite of the risk of the radiation.

CT is the gold standard for the evaluation of abdominal pathologies, especially for small bowel and colonic pathologies. CT can demonstrate the location of the caecum and ascending colon with the method that follows the intestinal segments sequentially from proximal to distal (stomach to anus) or distal to proximal anus to stomach). CT can also help in differentiation of small bowel from large bowel by demonstrating valvulae conniventes or haustrae. CT, in the case of malrotation, will also help in diagnosis by showing the relationship between the superior mesenteric artery and vein. In our first case, the ascending colon and caecum is located in left upper quadrant and inflamed appendix extended inferomedially from caecum to umbilicus. In the right lower quadrant, only small bowel segments were located. In our second case, caecum located more inferomedially from its normal anatomic position. The base of the appendix is located retrovesically; appendix extended laterally and its free end located near the iliac vessels.

In conclusion, in patients with abdominal pain, if there are atypical clinical features, imaging studies especially CT are helpful in identifying the location of caecum and appendix and avoiding from misdiagnoses.
